# Application and investigation of a bound for outcome reporting bias

**DOI:** 10.1186/1745-6215-8-9

**Published:** 2007-03-06

**Authors:** Paula R Williamson, Carrol Gamble

**Affiliations:** 1 Centre for Medical Statistics and Health Evaluation, Shelley's Cottage, Brownlow Street, University of Liverpool, L69 3GS, UK

## Abstract

**Background:**

Direct empirical evidence for the existence of outcome reporting bias is accumulating and this source of bias is recognised as a potential threat to the validity of meta-analysis of randomised clinical trials.

**Methods:**

A method for calculating the maximum bias in a meta-analysis due to publication bias is adapted for the setting where within-study selective non-reporting of outcomes is suspected, and compared to the alternative approach of missing data imputation. The properties of both methods are investigated in realistic small sample situations.

**Results:**

The results suggest that the adapted Copas and Jackson approach is the preferred method for reviewers to apply as an initial assessment of robustness to within-study selective non-reporting.

**Conclusion:**

The Copas and Jackson approach is a useful method for systematic reviewers to apply to assess robustness to outcome reporting bias.

## Background

Publication bias, whereby an entire study is either published or not depending on its results, is recognised as a potential threat to the validity of any meta-analysis. Empirical research suggests that published work is more than twice as likely to be statistically significant (p < 0.05) than unpublished research [1]. Several different methods for the identification of and adjustment for this source of bias are available [2]. More recently, asymptotic results have been presented that allow the meta-analyst to estimate the maximum bias possible in the treatment effect estimate assuming a certain number of trials have not been published [3]. Although the method is proposed as a sensitivity analysis, by varying the number of trials assumed to be missing, rather than a correction for bias, it is still important to investigate how well the result holds since meta-analyses typically include only 5–10 studies. Copas and Jackson suggest bootstrapping as a method for doing this however this is unlikely to be implemented by systematic reviewers unless made available in meta-analysis software.

Within-study selective reporting, or outcome reporting bias, has been defined as 'the selection of a subset of the original variables recorded for inclusion in publication of trials' [4]. This may include the situation where only one of several outcomes measuring similar things may be reported, e.g. weight gain at three or six months, or it may relate to a subtly different selection issue where a particular outcome is not reported on the basis of the results obtained [5]. Direct empirical evidence for the existence of such bias is accumulating [6,7]. In a meta-analysis it is often the case that a total number of eligible studies *k *are identified but only *n *(*k *> *n*) report the data of interest. The reviewer needs to examine the remaining (*k-n*) studies and try to establish whether the particular outcome has been collected but not reported. This should ideally involve contact with the original trialists which may result in missing data being made available or it may confirm that the outcome data were not recorded [8]. However it is likely that in a subset of these studies, *m *(≤ *k-n*) say, no such information is forthcoming. If the level of suspicion that selective non-reporting has occurred in these *m *studies is high, it has been recommended that a sensitivity analysis be undertaken assuming such bias has occurred [8].

We have previously proposed and applied a method for imputing data in this setting when the outcome of interest is binary and each trial compares two treatments [8]. Applying the Copas and Jackson bound with known *m *is easier to compute than the imputation method and therefore potentially very useful as an initial sensitivity analysis to assess robustness to extreme within-study selective non-reporting. In this paper we present a simulation study undertaken to assess how the two methods perform when bias arises as a result of various realistic suppression models.

## Methods

In sections 2.1 and 2.2 we describe the two methods of bias adjustment based on theoretical considerations and imputation respectively. Each method is applied to five meta-analyses from a cohort previously described [8]. Some discrepancies between results from the two methods were noted. In section 2.3 we describe a simulation study undertaken to gain an understanding of how the two methods perform in realistic meta-analysis and trial suppression model settings.

### 2.1 Maximum bias bound

For a given number of unpublished studies, *m*, the maximum bias, *b*, in the treatment effect estimate is given by the formula

|b|≤n+mnφ{Φ−1(nn+m)}∑i=1nσi−1∑i=1nσi−2     (2.1)
 MathType@MTEF@5@5@+=feaafiart1ev1aaatCvAUfKttLearuWrP9MDH5MBPbIqV92AaeXatLxBI9gBaebbnrfifHhDYfgasaacH8akY=wiFfYdH8Gipec8Eeeu0xXdbba9frFj0=OqFfea0dXdd9vqai=hGuQ8kuc9pgc9s8qqaq=dirpe0xb9q8qiLsFr0=vr0=vr0dc8meaabaqaciaacaGaaeqabaqabeGadaaakeaadaabdaqaaiabdkgaIbGaay5bSlaawIa7aiabgsMiJoaalaaabaGaemOBa4Maey4kaSIaemyBa0gabaGaemOBa4gaaGGaciab=z8aMnaacmqabaGaeuOPdy0aaWbaaSqabeaacqGHsislcqaIXaqmaaGcdaqadaqaamaalaaabaGaemOBa4gabaGaemOBa4Maey4kaSIaemyBa0gaaaGaayjkaiaawMcaaaGaay5Eaiaaw2haamaalaaabaWaaabCaeaacqWFdpWCdaqhaaWcbaGaemyAaKgabaGaeyOeI0IaeGymaedaaaqaaiabdMgaPjabg2da9iabigdaXaqaaiabd6gaUbqdcqGHris5aaGcbaWaaabCaeaacqWFdpWCdaqhaaWcbaGaemyAaKgabaGaeyOeI0IaeGOmaidaaaqaaiabdMgaPjabg2da9iabigdaXaqaaiabd6gaUbqdcqGHris5aaaakiaaxMaacaWLjaWaaeWaaeaacqaIYaGmcqGGUaGlcqaIXaqmaiaawIcacaGLPaaaaaa@63F0@

where *n *is the number of studies reporting data and σ_i _is the standard error of the treatment effect estimate in study *i *[3]. The approach assumes that larger studies (with small standard error) are more likely to be published than smaller studies (with larger standard error). The number of unpublished studies is usually unknown and thus sensitivity analysis, varying the unknown number of unpublished studies, *m*, is recommended. In the context of within-study selective non-reporting, the number of studies found to be eligible, where the outcome is known or suspected to have been measured but no results were presented, is known. If we take m to equal this number, (2.1) can be used to assess the robustness to this form of bias. A pooled effect estimate is first calculated from the *n *studies reporting data and either a fixed or random effects model as appropriate The bias-adjusted estimate is calculated by adding the value of this bound to the pooled effect estimate. Either +b or -b is added depending on the direction of effect such that the estimate is moved closer to the null.

### 2.2 Imputation of missing data

The maximum possible value for the pooled log odds ratio was also estimated by imputing missing data, specifically the number of events in each treatment group, for each of the *m *studies under the extreme assumption that the reason they are missing is because the two tailed p-value from the trial was greater than 0.05, and then combining this with observed data [8]. All possible imputation combinations are enumerated and in turn are pooled with the available data from the other *n *studies to produce a histogram of the meta-analysis estimates. For those examples where τ^2 ^> 0, random effects estimates were also calculated incorporating the between-study variance estimated from the observed data. The maximum imputation-adjusted estimate for the pooled log odds ratio is taken from the distribution of all possible values under this imputation method. An assessment is then made as to whether the inference is robust to the extreme value of the histogram or not. This approach is attractive since establishing robustness to the most extreme scenario avoids the need to undertake more complex analyses.

The imputation can be constrained by using information, either in the report or from clinical knowledge, to reduce the range of the possible number of events in a particular treatment group. For example, reported data from an associated outcome such as cancer-specific mortality may be taken as the lower limit for all-cause mortality. Unconstrained data were imputed for all examples. Constrained data were imputed for two reviews as follows [8]. In the cancer example, information from trial reports on subgroups and infection-related mortality was used to limit the range of the possible number of overall deaths. In the immunoglobulin review, by definition the number of serious infections had to be at least equal to the number of events reported for either sepsis or death from infection, providing a lower bound.

### 2.3 Simulation study

Data for the treatment group were simulated from a Bin(N, pt) distribution, for the control group from a Bin(N, pc) distribution. The choice of pt and pc determines the log odds ratio, log(OR). The sample size, N, was varied across trials according to a normal distribution with mean *N*_*μ *_and standard deviation υ * *N*_*μ *_where *υ *could take values between 0 and 1 with increasing values providing greater sample size variability between trials within a meta-analysis. A lower limit for N was applied such that values of N could not be less than 10 by setting any values generated less than this equal to 10. Data were simulated from *k *= *n *+*m *trials.

The true relative treatment effect was varied with values of the odds ratio taken as 1, 0.7, 0.5, and 0.3 by setting pt equal to 0.25 and varying the value of pc. The total number of trials before suppression, *k *= *n *+ *m*, was taken as 5,6,7,8,10, and 15 with the value of *m *allowed to take values equal to but not greater than *n*. Trial size was varied by taking values for *N*_*μ *_of 25, 50, 75, 100, 150, 200, and 300 and *υ *of 0.05, 0.15, and 0.35.

Three selection mechanisms have been considered: standard errors, one- and two-tailed p-values. Suppression on the basis of one- and two-tailed p-values and standard errors is not the same as the selection mechanism resulting in the Copas and Jackson bias bound being attained. However our objective in this paper is to assess how robust the methods are to realistic suppression models. A selection mechanism based on standard errors alone will not systematically bias the results of a meta-analysis, although there will be some loss of precision. Selection mechanisms based on one and two-tailed p-values will provide similar results the further the true effect is from the null. However there will be differences in the studies suppressed and resulting bias when the true odds ratio is close to or equal to one. In this situation suppression based on one-tailed p-values will systematically bias the results in one direction by suppressing a corner of the funnel plot producing an asymmetrical funnel, while suppression using two tailed p-values will produce a hollow funnel similar to suppression based on standard errors and will not systematically bias results in a particular direction. Therefore of the three selection mechanisms one-tailed p-value selection will produce the strongest bias and is arguably the most realistic suppression mechanism as it allows for differential selection according to the direction of the treatment effect.

The true bias in the treatment effect estimate was calculated as the difference between the pooled log(OR) from all *n *+ *m *trials (unsuppressed) and the pooled log(OR) based on the selected trials. The Copas and Jackson bias bound was calculated using (2.1) above. For each simulated dataset, the difference between the pooled log(OR) from all *n *+ *m *trials (unsuppressed) and the Copas and Jackson adjusted estimate was calculated. This simulation exercise was repeated 10,000 times for each combination of parameters pt, pc, *N*_*μ*_, and *υ*. For settings where the alternative method of imputation may be considered, namely small study sizes and few trials not reporting the data of interest, results were also obtained after adjustment via the imputation method for comparison. In settings where larger numbers of larger studies are missing, imputation was not considered due to the computational time involved [8].

## Results

### 3.1 Results for real examples

Table 1 shows the pooled estimates for each example following bias adjustment via both imputation and the Copas and Jackson approach (original data available on request from the first author). There are some differences in the results from the two methods. In the cancer meta-analysis, there is both a small number of studies presenting results in the trial publication and a large variability in the size of the standard errors of the reported effect estimates, and the difference between the results from the two methods of adjustment is marked. The albumin example includes a larger number of trials similar in size however, and the bias-adjusted estimates are almost identical.

**Table 1:** Estimates of bias-adjusted pooled effect in five meta-analysis case studies. FE: fixed effects, RE: random effects.

**Example**	**Cancer**	**Immunoglobulin**	**Prostaglandin**	**Albumin**	**Asthma**
Original pooled logOR:					
FE	-0.360	-0.204	-0.071	0.366	-1.258
RE		-0.311	-0.142		-1.376
Tau-squared	0	0.111	0.200	0	0.403
N	5	12	18	18	17
(Range of number of participants in trials)	(44 to 733)	(66 to 2416)	(20 to 2517)	(10 to 141)	(9 to 50)
M	4	3	1	2	1
(Number of participants per trial)	(67,91,146, 750)	(20,111,235)	(60)	(16,28)	(35)
Copas-adjusted estimate:					
FE	-0.045	-0.133	-0.033	0.591	-1.153
RE		-0.135	-0.050		-1.241
Maximum imputation-adjusted estimate:					
FE (constrained)	0.229	-0.111	-0.027	0.589	-1.034
FE (unconstrained)	0.269	-0.110	-0.027	0.589	-1.034
RE		-0.154	-0.049		-1.160

The difference between the two estimates is most marked in the cancer example with the imputation approach resulting in a much greater bias adjustment. Clearly the smallest studies are not suppressed here but neither are they in the other examples where the methods gave more similar results. Motivated by this example in particular, our aim in the rest of this paper is to understand how the two methods perform in a variety of settings via a simulation study.

### 3.2 Simulation study

**Figure 1:**
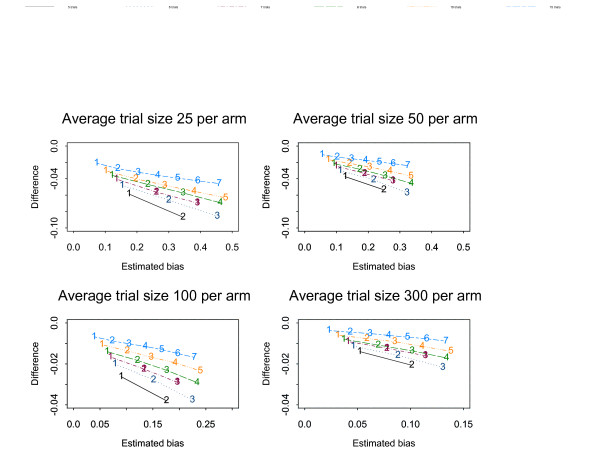
The numbers on each line represent the number of studies suppressed. The y-axis is the difference between the estimated bias and the Copas and Jackson bias bound. Simulation results: OR = 1, selection based on one tailed p-values, trial size variability = 0.05.

**Figure 2:**
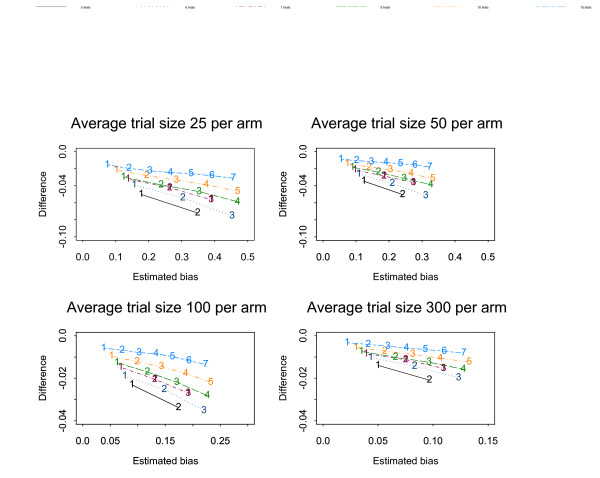
The numbers on each line represent the number of studies suppressed. The y-axis is the difference between the estimated bias and the Copas and Jackson bias bound. Simulation results: OR = 0.7, selection based on one tailed p-values, trial size variability = 0.05.

**Figure 3:**
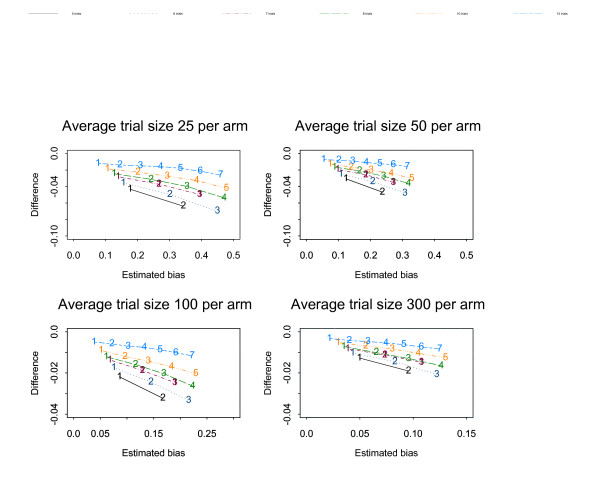
The numbers on each line represent the number of studies suppressed. The y-axis is the difference between the estimated bias and the Copas and Jackson bias bound. Simulation results: OR = 0.5, selection based on one tailed p-values, trial size variability = 0.05.

**Figure 4:**
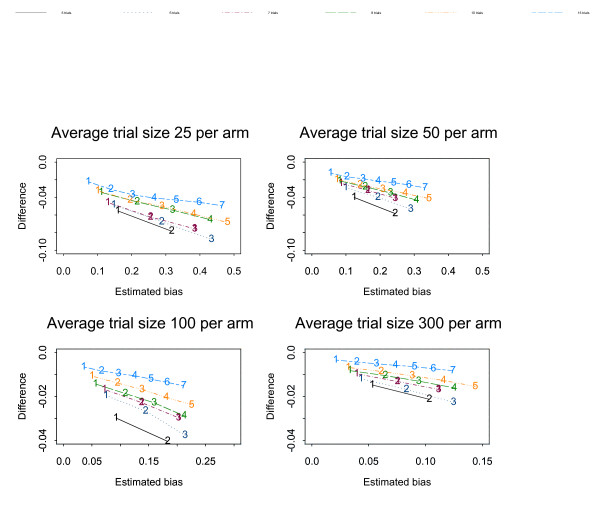
The numbers on each line represent the number of studies suppressed. The y-axis is the difference between the estimated bias and the Copas and Jackson bias bound. Simulation results: OR = 1, selection based on one tailed p-values, trial size variability = 0.35.

**Figure 5:**
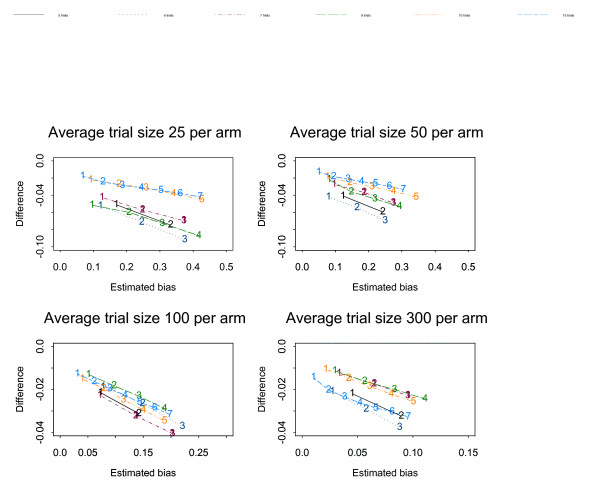
The numbers on each line represent the number of studies suppressed. The y-axis is the difference between the estimated bias and the Copas and Jackson bias bound. Simulation results: OR = 0.7, selection based on one tailed p-values, trial size variability = 0.35.

**Figure 6:**
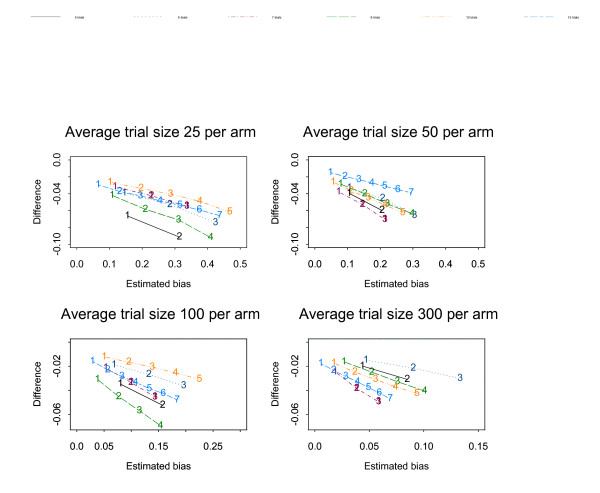
The numbers on each line represent the number of studies suppressed. The y-axis is the difference between the estimated bias and the Copas and Jackson bias bound. Simulation results: OR = 0.5, selection based on one tailed p-values, trial size variability = 0.35.

Figures 1, 2, 3, 4, 5, 6 show the results of the simulation study for the effects of increasing the treatment effect size and variability in trial size around the average shown when suppression is based on the one-tailed p-value. The median difference between the true bias and the Copas and Jackson bias bound is plotted against the median true bias, where median values have been found from the distribution of values across the 10,000 simulated datasets.

The plots show that, under the selection model based on one-tailed p-values, the bias increases as the number of suppressed trials increases, and the Copas and Jackson bias bound overestimates the bias, as expected. This conservatism increases as the number of suppressed trials increases. These results were similar across all three suppression models. Importantly these results provide information on the degree of overestimation in realistic settings.

Table 2 shows the 2.5th, 50th and 97.5th centiles of the distribution of the difference between the pooled log(OR) from all *n *+ *m *trials (unsuppressed) and the bias-adjusted estimates from the two methods for selected simulation parameters. The number of trials has been chosen to be five or 10 since most meta-analyses fall into this range. The results indicate that for a meta-analysis that includes a small number of small trials, the bias could be substantially underestimated in a proportion of cases. This was not the case however for selection based on standard errors. For suppression based on one- and two-tailed p-values, the accuracy of the Copas and Jackson estimate increased with increasing number of trials, larger trial size, decreased trial size variability and larger treatment effects. For suppression based on standard errors, the accuracy of the Copas and Jackson estimate increased with increasing number of trials, larger trial size, increased trial size variability and smaller treatment effects.

**Table 2:** Selected simulation study results. C&J: Copas and Jackson bias bound approach

**Odds ratio**	**Sample size variability parameter**	**Mean trial size**	**n**	**m**	**True bias: median**^1^	**Difference between unsuppressed pooled and C&J-adjusted: median (2.5^th^, 97.5^th^) centiles**	**Difference between unsuppressed pooled and maximum imputation-adjusted: median (2.5^th^, 97.5^th^) centiles**
1	0.05	25	4	1	0.175	-0.059 (-0.170, 0.117)	-0.142 (-0.334, 0.016)
1	0.05	25	3	2	0.345	-0.087 (-0.293, 0.228)	-0.323 (-0.653, -0.053)
1	0.05	300	4	1	0.052	-0.014 (-0.048, 0.043)	
1	0.05	300	3	2	0.101	-0.021 (-0.084, 0.071)	
1	0.35	25	4	1	0.159	-0.055 (-0.161, 0.121)	-0.146 (-0.371, 0.030)
1	0.35	25	3	2	0.317	-0.079 (-0.277, 0.220)	-0.310 (-0.661, -0.051)
1	0.35	300	4	1	0.053	-0.015 (-0.049, 0.048)	
1	0.35	300	3	2	0.103	-0.021 (-0.087, 0.077)	
0.5	0.05	25	4	1	0.176	-0.043 (-0.159, 0.153)	-0.236 (-0.437, -0.060)
0.5	0.05	25	3	2	0.343	-0.064 (-0.280, 0.277)	-0.593 (-0.943, -0.300)
0.5	0.05	300	4	1	0.049	-0.013 (-0.047, 0.045)	
0.5	0.05	300	3	2	0.096	-0.019 (-0.082, 0.071)	
0.5	0.35	25	4	1	0.155	-0.066 (-0.199, 0.200)	-0.358 (-0.650, -0.150)
0.5	0.35	25	3	2	0.314	-0.091 (-0.311, 0.307)	-0.546 (-0.946, -0.245)
0.5	0.35	300	4	1	0.044	-0.020 (-0.060, 0.048)	
0.5	0.35	300	3	2	0.085	-0.031 (-0.104, 0.075)	
1	0.05	25	9	1	0.101	-0.030 (-0.080, 0.039)	-0.056 (-0.125, 0.013)
1	0.05	25	5	5	0.479	-0.063 (-0.270, 0.198)	
1	0.05	300	9	1	0.031	-0.006 (-0.022, 0.019)	
1	0.05	300	5	5	0.138	-0.014 (-0.075, 0.063)	
1	0.35	25	9	1	0.101	-0.033 (-0.086, 0.059)	-0.077 (-0.182, 0.011)
1	0.35	25	5	5	0.481	-0.068 (-0.274, 0.221)	
1	0.35	300	9	1	0.032	-0.007 (-0.025, 0.025)	
1	0.35	300	5	5	0.144	-0.015 (-0.080, 0.076)	
0.5	0.05	25	9	1	0.105	-0.019 (-0.072, 0.065)	-0.126 (-0.206, -0.045)
0.5	0.05	25	5	5	0.477	-0.042 (-0.250, 0.239)	
0.5	0.05	300	9	1	0.030	-0.005 (-0.021, 0.019)	
0.5	0.05	300	5	5	0.130	-0.013 (-0.072, 0.063)	
0.5	0.35	25	9	1	0.102	-0.026 (-0.086, 0.083)	-0.027 (-0.096, 0.071)
0.5	0.35	25	5	5	0.467	-0.062 (-0.281, 0.261)	
0.5	0.35	300	9	1	0.017	-0.017 (-0.036, 0.020)	
0.5	0.35	300	5	5	0.094	-0.043 (-0.110, 0.046)	

^1 ^calculated as the median of the distribution of values across simulations for the difference between the pooled log(OR) from all *n *+ *m *trials and the pooled log(OR) based on the selected trials

The simulation results demonstrate that the imputation method leads to systematically greater over-adjustment for bias compared to the Copas and Jackson method. We believe this to be the explanation for the difference between the results of the two methods in the cancer example shown in Table 1.

## Discussion

In this paper we advocate that robustness of the meta-analysis to outcome reporting bias be assessed where there is a high level of suspicion that within-study selective reporting has occurred. We recommend sensitivity analysis rather than adjustment, since correction for bias is impossible without knowledge of the exact selection mechanism operating. Understanding selection bias is made all the more difficult when one recognises that the process may vary across different fields, for example mechanisms operating in genetic epidemiology may differ from clinical trials [9].

In some instances it may be obvious that an outcome was measured even if not reported given the other outcomes included in a trial publication. For example, if cause-specific mortality is reported then overall mortality must have been measured, even if not reported. In other situations it may be that a battery of tests or measurements are usually undertaken together, for example systolic and diastolic blood pressure, such that if one outcome is reported but another is not, one should be suspicious that the latter may have been selectively not reported. However it is probable that it will often be difficult to assess whether the outcome was measured and judgment will be required. The ORBIT (Outcome Reporting Bias In Trials) project, funded by the UK Medical Research Council, will attempt to further our understanding of the processes resulting in selective outcome reporting through interviews with clinical trialists. The sensitivity and specificity of a method for assessing outcome reporting bias in a trial will be estimated by comparing the assessment based on all trial reports with the information obtained directly from trialists.

Researchers often rely on the shape of funnel plots to detect publication bias however empirical studies suggest that this may be misleading [10]. Tests for asymmetry in a funnel plot, including that of Egger, have low power in typical meta-analyses involving 5–10 trials [11]. It may be of interest to undertake such a test, and a significant result may be taken as evidence of asymmetry, however the sources of bias, be they methodological quality or outcome reporting bias or some other small study effects, need further investigation. We prefer not to rely on a non-significant result as indicative of a lack of bias when evidence from clinical opinion or the trial paper, e.g. p-value for the outcome reported to be >0.05, clearly raises our level of suspicion.

Assessing the robustness of a meta-analysis to extreme within-study selective non-reporting is a useful first stage. Imputation of missing data has the advantage that it can be constrained by information given in the report. However the disadvantages include the programming and computational time required as well as the limitation to binary outcomes. The approach suggested in this paper based on the method of Copas and Jackson has the advantage of being simple to compute. However information given in the report cannot be used to provide a tighter bound.

The simulation results provide useful information for practical meta-analysts. The approach taking the extreme estimate under imputation has been shown to work poorly by severe over-adjustment. The Copas and Jackson adjustment works well for most cases investigated under a variety of true suppression models. In situations where the treatment effect is small, trial sizes are small and/or variable, the number of studies with available data is small and the number with missing outcome data large, the approach was found to be less accurate. However, in these situations the adjustment is conservative, and hence a meta-analysis which is found to be robust after this degree of adjustment can be considered to be robust to this form of bias.

There are several issues in this work worthy of further development and investigation. Firstly, the application of the method to the problem of within-study selective non-reporting makes no allowance for the possibility that there are also further studies that may have collected data on the outcome of interest that are simply not known about. As with other papers in this area [8,12], this should be viewed as an initial exploratory analysis. If the meta-analysis is not robust to within-study selective reporting bias this needs to be recognised. If the results are robust, further work should be undertaken regarding bias due to unpublished studies. Further work is needed to evaluate this two-stage approach and also to consider methods to allow for both within- and between-study selective reporting simultaneously. Secondly, the method has only been investigated for trial settings in the absence of heterogeneity of treatment effect across studies. Thirdly, in their work related to between-study selection bias, Copas and Jackson comment that "In reality, significance is not the only factor taken into account by editors and referees, or even by authors in deciding whether to write up their study and submit the article in the first place". It may be however that statistical significance plays a larger part in the decision about which outcomes to present within a report. Further work is needed to understand the selection mechanisms operating, and there may be more than one in any particular setting, in order to better inform statistical modelling in this area.

## Conclusion

The Copas and Jackson approach is a useful method for reviewers to apply to assess robustness to within-study selective non-reporting. A question for further research is whether an improved method can be developed in situations where the trials are small or there are few trials with missing outcome data.

## Competing interests

The author(s) declare that they have no competing interests.

## Authors' contributions

PW conceived of the study, designed and coordinated the study, and drafted the manuscript. CG participated in the design of the study, carried out the simulation study and performed the statistical analysis. Both authors read and approved the final manuscript.
